# Changes in Pulmonary Microenvironment Aids Lung Metastasis of Breast Cancer

**DOI:** 10.3389/fonc.2022.860932

**Published:** 2022-05-26

**Authors:** Meimei Wu, Yanfang Liang, Xin Zhang

**Affiliations:** ^1^ Clinical Experimental Center, Jiangmen Key Laboratory of Clinical Biobanks and Translational Research, Jiangmen Central Hospital, Jiangmen, China; ^2^ Department of Pathology, Dongguan Hospital Affiliated to Jinan University, Binhaiwan Central Hospital of Dongguan, Dongguan, China; ^3^ Dongguan Key Laboratory of Medical Bioactive Molecular Developmental and Translational Research, Guangdong Provincial Key Laboratory of Medical Molecular Diagnostics, Guangdong Medical University, Dongguan, China; ^4^ Collaborative Innovation Center for Antitumor Active Substance Research and Development, Guangdong Medical University, Zhanjiang, China

**Keywords:** breast cancer, lung metastasis, pulmonary microenvironment, inflammation, angiogenesis, extracellular matrix remodeling, neutrophils

## Abstract

Breast cancer has become the most common malignant disease in the world according to the International Agency for Research on Cancer (IARC), and the most critical cause of death is distant metastasis. The lung is the extremely common visceral site for breast cancer metastasis. Lung metastasis of breast cancer is not only dependent on the invasive ability of the tumor itself, but also closely relates to the pulmonary microenvironment. In the progression of breast cancer, the formation of specific microenvironment in lungs can provide suitable conditions for the metastasis of breast cancer. Pulmonary inflammatory response, angiogenesis, extracellular matrix remodeling, some chemotherapeutic agents and so on all play important roles in the formation of the pulmonary microenvironment. This review highlights recent findings regarding the alterations of pulmonary microenvironment in lung metastasis of breast cancer, with a focus on various cells and acellular components.

## Introduction

**Figure 1:**
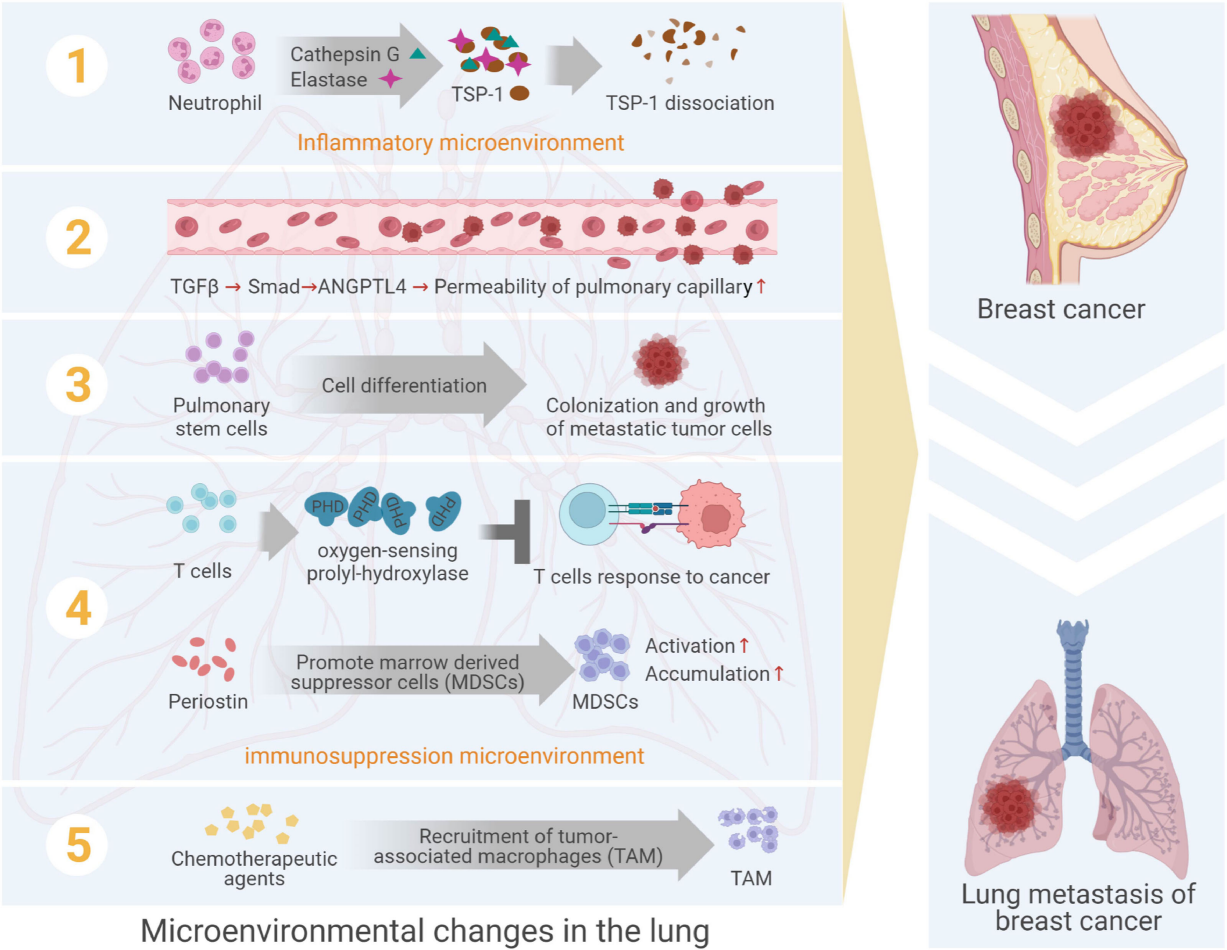
The formation of pulmonary microenvironment in lung metastasis from breast cancer. The formation of sites adapted to the survival of metastatic cancer cells is regulated by acellular components and a variety of cells, including neutrophils, vascular endothelial cells, stem cell-like lung cells, immune cells, etc.

Among female cancers, breast cancer had awfully high morbidity and mortality (1, 2), and was highly malignant, poor prognosis and prone to local recurrence and distant metastasis (3–5). Lung metastasis was a very common distant metastasis of breast cancer, the incidence of which was about 21%~32% (6), and the prognosis of patients with lung metastasis was greatly poor, with a median survival of only 25 months (7). In the process of lung metastasis of breast cancer, the breast cancer cells would undergo several steps, such as leaving the primary lesion, invading the circulatory system, reaching the lung tissues, colonizing in lungs and forming clinically visible metastasis (8, 9). In this series of activities, metastatic breast cancer cells were considered as “seeds”, and the microenvironment in metastatic niche was considered as “soil”. Besides, the formation of metastatic niche was the result of the interaction between “seed” and “soil”. However, previous studies focused on the molecular and functional changes of metastatic cancer cells themselves, ignoring the induction or interference of the microenvironment in the process of metastasis. In recent years, the study of lung microenvironment of breast cancer metastasis showed that the dynamic changes of “soil” in this process were as important as the ability of “seed” itself. Therefore, this review mainly discussed the role of various cells, particularly neutrophils, and acellular elements in pulmonary microenvironment targeting breast cancer lung metastasis (**Figure 1**).

## Neutrophils in the Pulmonary Microenvironment

By the 1860s, Virchow (10) had found a large number of leukocytes in tumor tissue and established a link between inflammation and cancer. With the further research, the close relationship between inflammation and cancer had gradually been confirmed (10–12). Existing studies displayed that the lung was a relatively open organ of the body to the external environment and was susceptible to various external stimuli to induce inflammation (13–15), and the lung inflammation enhanced tumor metastatic outgrowth in lungs (16). Bacterial lipopolysaccharide (LPS) and Nicotine in cigarettes were well-characterized inducer of lung inflammation (13, 15, 17).

LPS-mediated and cigarette smoke-induced lung inflammation were closely related to breast cancer metastasis to the lungs (18, 19), which was marked by increased neutrophils influx and up-regulation of pro-inflammatory cytokines (16, 20). Also, smoking promoted lung metastasis of breast cancer precisely because lungs continued to be exposed to nicotine, which generated inflammatory microenvironment in lungs (20). Pulmonary inflammatory microenvironment would result in the influx of a vast number of activated neutrophils and the formation of appropriate tumor metastasis niche in lungs. Furthermore, some tumor-promoting neutrophils released lipocalin 2 (LCN2) protein activated by STAT3 to induce tumor metastatic growth (20). Thus, neutrophils might play an important role in pulmonary metastatic microenvironment.

### Neutrophil-Dependent Inflammatory Microenvironment in Lungs

Neutrophils were the major circulating leukocytes in human body. Besides, neutrophils were recruited as the first line of defense in the immune response to fight off acute inflammation (21). If pathogens broke through the physical barrier formed by the skin and mucous membranes to enter the tissues, pathogens at the site of infection and macrophages produced signals to activate endothelial cells, which captured circulating neutrophils and induced them to cross the endothelium to bind to the pathogen (21–23). After migration from the blood to the tissues, neutrophil immune function was enhanced, while activated neutrophils produced specific active substances that attracted more inflammatory cells including neutrophils, macrophages and T cells to the site of inflammation and regulate their activity (21, 24). The activated neutrophils delivered lethal blows to pathogens through various mechanisms such as degranulation and release of active proteins. Nevertheless, hyperactivated neutrophils often lead to significant damage to normal tissue (21, 25).

Lung tissues were the main site where many pathogenic microorganisms invaded the body, thus lung tissues were particularly dependent on the surveillance of innate immune cells dominated by neutrophils (26, 27). Besides, lungs were the major neutrophil reservoir. When the lungs were infected, neutrophils leaked from pulmonary circulating capillaries into the lungs, and the neutrophils then activated and engulfed pathogens and released various active substances (28). Neutrophils killed pathogens in lungs by two main mechanisms: oxidative and non-oxidative. The oxidative pathway was the primary mode of pathogen killing by neutrophils through the production of nicotinamide adenine dinucleotide phosphate (NADPH) oxidase system (29). Non-oxidative pathways included various antimicrobial proteins and proteases, such as neutrophil elastase (NE), myeloperoxidase (MPO), alkaline phosphatase (ALP), defensins, lysozyme and neutrophil extracellular traps (NETs), which promoted neutrophils phagocytosis of pathogens, neutrophils migration, and regulated the inflammatory response (30–32).

Although neutrophils played an important role in fighting infections with lung pathogens such as bacteria, fungi and viruses, continuous neutrophil infiltration could facilitate lung metastasis. Neutrophils in lungs could be “hijacked” by cancer cells and helped them spread into lungs.

### Molecular Mechanisms of Pulmonary Neutrophils That Promote Lung Metastasis of Breast Cancer

Neutrophils were recruited in the niche and altered their polarization state in the tumor-bearing host, switching from suppressing to promoting roles in tumor metastasis (33, 34). Neutrophils could elicit a metastatic inflammatory microenvironment by suppressing innate and adaptive anti-tumor immunity (35–37). With the increased focus on neutrophils accelerating lung metastasis in recent years, there was accumulating evidence indicating that neutrophils in lungs played a key role in the formation of the metastatic niche in lungs. Signaling factors derived from cancer cells activate neutrophils stored in lungs or recruited neutrophils to migrate into lungs, thereby neutrophils promoted tumor metastasis into lungs and colonize in lungs.

It was found that tumor exosomal RNAs promoted lung metastatic niche formation by activating alveolar epithelial Toll-like receptor 3 (TLR3) to recruit neutrophils (38). TLR3 in host alveolar epithelial cells was critical for neutrophil recruitment and niche formation before lung metastasis (38, 39). In mice with TLR3 deficiency, the incidence of lung metastasis was significantly reduced, and the survival of tumor-bearing mice was significantly prolonged (38). TLR3 was highly expressed in lung epithelial cells (40), and the lung epithelial TLR3 pathway was activated on exposure to virus, fungus, and even cigarettes (41, 42). Interestingly, high epithelial TLR3 expression was correlated with smoke-induced chronic inflammation in lungs (43), which might explain why smoking facilitated lung metastasis of breast cancer as mentioned above.

Liu et al. revealed that the natural immune receptor TLR3 expressed by type II lung epithelial cells could identify the exosomal RNA secreted by tumor into the blood circulation (44–46), thus triggering the expressions of chemokines (CXCL1, CXCL2, CXCL5, and CXCL12) and recruiting neutrophils into the lungs (38). Neutrophils in the bone marrow showed no significant differences in the mice with or without tumor inoculation. It was known that chemokine and its receptor contributed to neutrophil mobilization and recruitment. Moreover, Liu et al. discovered a marked increase in the level of chemokines (CXCL1, CXCL2, CXCL5, and CXCL12) in serum and bronchoalveolar lavage fluid after administration of exosomes, at which time there were no cancer cells in lungs (38). Accordingly, neutrophils might accumulate in the pre-metastatic lung induced by tumor exosomal RNAs.

Additionally, Xiao et al. showed that breast cancer cells secreted cathepsin C exciting neutrophils in lungs and recruiting more neutrophils into lungs to promote lung metastasis of breast cancer (47). Cathepsin C, a significant lysosomal cysteine protease, mediated the maturation process of neutrophil serine proteases, and participated in the inflammation and immune regulation process associated with neutrophils (48). Cathepsin C, derived from breast cancer cells, promoted lung metastasis of breast cancer cells by regulating neutrophil infiltration and the formation of NETs in lungs metastasis microenvironment (47).

Mechanically, tumor-secreted cathepsin C promoted the maturation and release of IL-1β in pulmonary neutrophils by activating the neutrophils membrane localization proteinase 3 (PR3) through enzyme digestion (49, 50). PR3 up-regulated the neutrophils IL-1β/nuclear factor kappa B (NF-κB) signaling pathway and their well-known downstream targets of cytokines such as IL-6 and chemokine (C-C motif) ligand 3 (CCL3), resulting in attracting more neutrophils to migrate into the lungs (47, 51). At the same time, tumor-secreted cathepsin C also up-regulated neutrophils reactive oxygen species (ROS) levels through PR3-IL-1β-P38/mitogen-activated protein kinase (MAPK), inducing the formation of NETs to degrade the anti-tumor factor thrombospondin-1 (Tsp-1) in lungs metastasis microenvironment. NETs enhanced tumor progression by affecting the endothelium, platelets, and the extracellular matrix and even the tumor cells themselves (52–54). Besides, the production of ROS and the formation of NETs supported lung metastasis of breast cancer by downregulating the thrombospondin-1 (Tsp-1) (16, 47). Tsp-1 had been found to protect lung tissues from tumor development (55). Apart from ROS and NETs, Ser proteases, NE and cathepsin G released by neutrophils could target and destroy Tsp-1, thus promoting breast cancer lung metastasis (16, 56, 57).

Bioluminescent imaging analyses showed that cathepsin C-induced elevation of cancer cell signal in lungs was already prominent at the first week after intravenous inoculation, indicating a role of cathepsin C in the early stage of metastatic colonization (47). Nevertheless, within 7 days after inoculation, cathepsin C-enhanced cancer cells had been existed in lungs at the early time points. Thus, it was not confirmed that whether the cathepsin C released by cancer cells reached lungs *via* blood circulation prior to the migration of cancer cells to lungs and recruit neutrophils, or neutrophils were recruited by releasing cathepsin C after the migration of cancer cells to lungs and established the “congenial soil” for lung metastasis of breast cancer.

In addition to promoting breast cancer cell metastasis through NETs or ROS, neutrophil-derived leukotrienes also promoted cancer cell colonization. Leukotrienes were a natural chemical that promoted inflammation. When leukotrienes released in the body, it could cause constriction of the airways, muscle tightness, and excess mucus and fluid (58). It was known that cancer cells within tumors were heterogeneous and retained different tumorigenic potentials. Metastasis-initiating cells depended on a favorable microenvironment to efficiently growed at the distant site (59–61). Wculek et al. revealed that neutrophil-derived leukotrienes facilitated the colonization of lung tissues by selectively expanding the sub-pool of breast cancer cells that retained high tumorigenic potential (62). Consequently, genetic or pharmacological suppression of arachidonic acid 5-lipooxygenase (ALOX5), which was responsible for leukotrienes synthesis in neutrophils, eliminated the carcinogenic activity of neutrophils and inhibited metastasis formation.

In lungs, not only do pathogens induced the recruitment of neutrophils, but also tumor cells with pulmonary metastasis also released signals to recruit neutrophils. Concordantly, they all drived the recruitment of neutrophils to form the metastatic niche in lungs.

## Role of Other Cells in Lung Metastasis Microenvironment of Breast Cancer

The lung microenvironment was a very complex and changeable environment, and the formation of sites adapted to the survival of metastatic cancer cells was regulated by pulmonary inflammatory response, angiogenesis, immune response, extracellular matrix remodeling and so on. Among them, neutrophils played an extremely important part in lung inflammation. Beyond that, there were many other cells involved in the regulation of the reactions described above, including vascular endothelial cells, stem cell-like lung cells, immune cells, etc.

### Vascular Endothelial Cells in Lungs

The endothelial cells of the capillaries in lungs were tightly attached (63, 64), unlike the discontinuous endothelial cells in the vascular system of bone, which were easy for other cells to pass through (65). Breast cancer cells must first break through the barrier of capillaries in lungs to colonize and grow. It was found that TGF-β promoted the metastasis of estrogen receptor (ER)-negative breast cancer to the lungs by upregulating the expression of a cytokine called angiopoietin-like protein 4 (ANGPTL4) (66, 67), which blocked the contact between pulmonary vascular endothelial cells (68). Within the tumor microenvironment, TGF-β was produced by macrophages, mesenchymal cells and the cancer cells themselves (66). In breast carcinoma, selective losses of growth inhibitory responses often accrued through alterations downstream of Smad, leaving the rest of the TGFβ pathway operational and open to co-option for tumor progression advantage (69). Cancer cell-derived ANGPTL4 was a TGFβ target in breast cancer, which was involved in the regulation of cancer growth, metastasis and angiogenesis (70). ANGPTL4 was induced by TGF-β through Smad signal transduction pathway (71, 72), which resisted the tight connection and adherent connection of vascular endothelia, destroyed the integrity of capillary wall, increased the permeability of pulmonary capillary, as well as induced tumor cells to exudate from blood vessels and entered the lung tissues for colonization and growth (66, 67).

In other words, disruption of the tight junctions of vascular endothelial cells in lungs provided favorable conditions for the metastasis of cancer cells to the lung. Therefore, factors that damage pulmonary vascular endothelial cells should be taken into account. They might be used as a drug target to prevent lung metastasis of breast cancer, or as a predictive method for patients at high risk for lung metastasis of breast cancer, so that patients could be more closely monitored and more aggressively treated with drugs.

### Pulmonary Stem Cells

After successfully entering the lung tissues, breast cancer cells needed the assistance of lung cells for colonization and metastatic growth. Such a cluster of cells existed in lungs, alveolar type 1 (AT1) cells and AT2 cells. They mainly formed the lung’s respiratory units and alveoli (73, 74). During development, AT1 and AT2 cells arose directly from a bipotent progenitor, whereas after birth new AT1 cells derived from rare, self-renewing, long-lived, mature AT2 cells that produced slowly expanding clonal foci of alveolar renewal (74, 75). There was a switch after birth, when AT2 cells function as stem cells that contributed to alveolar renewal, repair and cancer progression (76, 77).

It was reported that breast cancer cells entered the lung tissues in the early phase, the function of AT2 cells as stem cell-like lung cells were enhanced, and various types of lung cells were differentiated, finally forming the metastatic niche suitable for the growth and colonization of breast cancer (78). Ombrato et al. designed a version of a secreted monomeric Cherry red fluorescent protein (mCherry) containing a modified lipo-permeable Transactivator of Transcription peptide (sLP-mCherry) (79, 80), to develop a labelling system where metastatic cancer cells directly identified their neighboring cells *in vivo (* 78). They engineered 4T1 breast cancer cells to express the sLP-mCherry protein alongside a canonical cell-retained Green Fluorescent Protein, which referred to as Labelling-4T1. Notably, when metastasis was formed, the number of mCherry+ niche cells in the tissue was still proportional to the growing metastatic cells. Cherry-niche epithelial cells had a higher proliferative activity compared to their normal lung counterpart. Furthermore, lineage-labelled AT2 cells showed no plasticity in co-culture with CD31+ cells. when exposed to cancer cells, lineage-labelled AT2 cells generated a notable amount of multi-lineage bronchioalveolar organoids, supporting the idea of a reprogramming activity driven by cancer cell-derived factors *ex vivo*. Concordantly, there were alveolar cell clusters with increased proliferative activity at the metastatic borders of human breast cancer lung metastasis, which suggested that the response of lung parenchyma to metastatic growth might occur in both mice and humans (78). However, it was not known how AT2 cells differentiate into large numbers of cells to support the growth of cancer cells.

### T Cells in Lungs

Cancer cells that had metastasized to targeted organs must also evade their immune response in order to colonize and grow. Metastasis of breast cancer cells to lungs and colonization of growth must also create an immune-permitting environment in lungs. T cells had an important impact in regulating immune function. Whereas effector T (Teff) cells promoted immune activation and would drive clearance of infections and cancer, regulatory T (Treg) cells, dependent upon the transcription factor forkhead box protein 3 (Foxp3), suppressed their function, preventing excessive autoimmune and allergic reactions (81, 82).

It was reported that expression of a protein called oxygen-sensing prolyl-hydroxylase (PHD) in T cells inhibited the immune response to cancer cells by interfering with T cells, allowing circulating cancer cells to colonize in lungs (83). In the normal body, the oxygen-sensitive PHD protein in lungs was designed to prevent the inflammatory responses that occurred when innocuous substances were inhaled during daily life (84, 85).

In breast cancer patients, however, the immunosuppressive effect of the oxygen-sensitive PHD protein in the highly oxygenated lung microenvironment opened the door to cancer cells and created a “fertile soil” for their growth. PHD proteins limited pulmonary type helper (Th)-1 responses, promoted CD4+ regulatory T (Treg) cell induction, and restrained CD8+ T cell effector function (83). Clever et al. engineered mice harboring a T cell-specific deletion of all three PHD proteins. *Cd4*-driven Cre recombinase expression resulted in significant reduction of *Egln1*, *Egln2*, and *Egln3* mRNA transcripts, which encoded PHD2, PHD1, and PHD3 proteins respectively, in CD4+, CD8+, and NKT T cells, but not in other lymphoid cell subsets. Upon tumor colonization, PHD proteins promoted Treg cell expansion and restrained IFN-γ-dependent clearance of tumors.

The researchers also found that using a drug inhibitor or knocking out the PHD protein in the T-cells of inbred mice stimulated an immune response to cancer cells and inhibited metastasis to the lungs (83). These findings provided a new immunological basis for explaining the susceptibility of breast cancer to lung metastasis.

### Myeloid-Derived Suppressor Cells (MDSCs) in Lungs

There was increasing evidence that MDSCs were key components of the pre-metastatic niche and played an important role in tumor cell implantation and metastasis by crafting pre-metastatic niches for proliferation, immunosuppression, and vascular remodeling (86, 87). More importantly, blocking the aggregation of these cells in target organs had been shown to prevent tumor metastasis (88). The initial events responsible for the metastasis process were the expansion of hematopoietic stem cells and progenitor cells in the bone marrow and their differentiation into MDSCs at the site of early metastasis (89, 90). MDSCs accumulated in the pre-metastatic niche also inhibited anti-tumor T cells through Arginase 1 and ROS production (91). In the presence of Breg cells, MDSCs produced more ROS and NO and became more suppressive to CD8+ T cells (92–94).

Carbonic anhydrase IX (CAIX) was one of the most highly expressed genes in the hypoxic environment of solid tumors. CAIX promoted intracellular pH buffering through extracellular CO2 hydration to produce bicarbonate and protons (95). Bicarbonate was transferred into cells by bicarbonate transporters to maintain alkaline intracellular pH conducive to cell survival, while the protons produced contributed to the acidification of the extracellular space and increased the migratory and invasive behavior of the tumor (96). Consistently, CAIX played an important role in the cellular invasion of breast cancer cells (97). Furthermore, CAIX was required for tumor growth and metastasis and maintenance of the stemness phenotype within the hypoxic niche of breast tumors (97, 98). It was found that hypoxia-induced CAIX expression in primary tumors derived from 4T1 breast cancer cells was needed for the production of chemokines and cytokines required for the mobilization of granulocytic MDSCs to a functional metastatic niche in a syngeneic preclinical model of spontaneous breast cancer lung metastasis (95). CAIX promoted the production of known soluble mediators of breast cancer metastasis, CXCL10, CCL5, and G-CSF, by hypoxic breast cancer cells (95). Besides, hypoxia-induced CAIX facilitated the activation of the NF-kB pathway causing the stimulation of G-CSF production to trigger MDSCs infiltration in the lung. Thus, during the early stages of breast cancer metastasis, CAIX promoted the development of the breast cancer lung metastatic niche *via* accelerating the production of G-CSF to recruit MDSCs.

### Macrophages in Lungs

Macrophages were the principal immune cells within the tumor microenvironment and were obligate partners for tumor cell migration and metastasis (99). Normal lung tissue contains an abundance of alveolar and interstitial macrophages (100). At metastatic sites a distinct population of metastasis associated macrophages promoted tumor cell extravasation, seeding and persistent growth (101). It was showed that breast tumor cell-released microparticles (T-MPs) from the primary tumor site played a critical role in the metastatic process. The T-MPs remodeled the lung parenchyma *via* a macrophage-dependent pathway to create an altered inflammatory and mechanical response to tumor cell invasion (102). Mechanistically, circulating T-MPs readily entered the lung parenchyma where they were taken up by local macrophages and induced CCL2 production. CCL2 recruited CD11b^+^Ly6C^high^ inflammatory monocytes to the lungs where they matured into F4/80^+^CD11b^+^Ly6C^-^ macrophages that not only produced IL6 but also triggered fibrin deposition. IL6 and the deposited fibrin facilitated the survival and growth of tumor-repopulating cells in the lungs by providing chemical and mechanical signals, respectively, thus setting the stage for lung metastasis of breast cancer (103).

## Effect of Acellular Components on Lung Metastasis Microenvironment of Breast Cancer

### Extracellular Matrix Proteins

For years the extracellular matrix proteins was mainly considered to serve as a scaffold, but now it was evident that the extracellular matrix proteins was a critical part of the tumor microenvironment (104). The extracellular matrix proteins regulated cancer cell behavior such as proliferation, adhesion, migration and differentiation (105) and its importance in cancer and metastasis had been extensively reviewed (106, 107).

Periostin was a multifunctional extracellular matrix protein, which was expressed by fibroblasts in the normal tissue and in the stroma of the primary tumor (59). Besides, periostin was closely related to the occurrence and development of a variety of tumors and provided an appropriate site for the metastatic growth of cancer cells by regulating the formation of neovascularization and immune regulation at the metastatic site (108, 109). It was reported that periostin protein derived from tumor fibroblasts determined the lung metastasis efficiency of breast cancer and the size of metastatic cancer. Wnt signaling activity in metastasis was abrogated in the absence of periostin. Wnt was known to control tumor stem cell maintenance in a variety of tissues (110, 111). Similarly, the levels of the general Wnt target gene *Axin2* in metastasis in wild-type hosts was higher than in mutant hosts. Periostin acted as a niche component that promoted tumor stem cell maintenance and metastatic colonization by augmenting Wnt signaling (59).

In addition, periostin protein played an important role in the metastasis microenvironment and perivascular niche (112). Periostin promoted the pulmonary accumulation of MDSCs during the early stage of breast tumor metastasis. Periostin deletion decreased neutrophil and monocytic cell populations in the bone marrow of mice and suppressed the accumulation of MDSCs to metastatic sites. Periostin-deficient MDSCs displayed reduced activation of ERK, AKT and STAT3 and periostin deficiency decreased the immunosuppressive functions of MDSCs during tumor progression. Moreover, lysyl oxidase contributed to periostin-promoted metastatic niche formation and tumor metastasis. However, the metastatic role of periostin was largely limited to ER-negative breast cancer patients (113).

### Energy and Nutrient Sources for Metastatic Cancer

The metastatic growth of cancer cells required not only the regulation of angiogenesis and immune regulation at the metastatic site, but also the supply of nutrients to satisfy its proliferation and growth (114, 115). It had been found that under the induction of signals released by tumor cells, neutrophils could not only infiltrate into lung tissues to play the role of immune regulation, but also stored a large amount of lipids (116, 117). Once tumor cells migrated to the lung, these lipids could be used as reserve food for tumor cells to promote their colonization and growth. These lipids were not inherent to the neutrophils, but were induced by CD140a+ mesenchymal cells once they reached the lungs (118). Lung mesenchymal cells significantly up-regulated the expression of neutrophil lipid droplet related genes, including *Hilpda*, *Cidec* and *Atgl (* 119, 120). These up-regulated triglyceride lipase inhibitors in turn inhibited ATGL enzyme activity, leading to the accumulation of triglycerides in pulmonary neutrophils (118). Neutrophil-specific knockout of *Atgl* enhanced lipid accumulation in cells and significantly promoted lung metastasis of breast cancer *in vivo*. In contrast, knockout of *Hilpda* genes in neutrophils reduced lipid storage and significantly inhibited metastatic colonization of breast tumors (118).

### Chemotherapy Drug

In the process of disease progression of cancer, chemotherapy drugs were like a double-edged sword. Although they had contributed to21 adjuvant chemotherapy after surgery or preoperative neoadjuvant chemotherapy, but some chemotherapeutic drugs promote metastasis while killing cancer cells (121, 122). It was found that breast cancer cells spread through recombinant TEK tyrosine kinase (TIE2)/Mammalian-enabled (MENA) pathway dependent infiltrating sites in cancer cells, namely, tumor microenvironment of metastasis (TMEM) (123, 124). Using fixed tissue and *in vivo* imaging of PyMT mice models and patient-derived xenografts (125), it was found that paclitaxel increased the density and activity of the TMEM site and MENA expression and promoted distant metastasis. Besides, in residual breast cancer patients treated with neoadjuvant paclitaxel after doxorubicin and cyclophosphamide, TMEM score and expression of mechanism related MENA^INV^ subtypes were increased, suggesting that although chemotherapy reduced tumor size, it increased the risk of metastasis and spread (126).

In addition, although paclitaxel was clearly beneficial in reducing tumor size, it increased the circulation of tumor cells in the blood, helping breast cancer cells escape from the primary focus, and directly acted on the lungs, altering the lung microenvironment and helping cancer cells colonize the lungs (127). In metastatic lung, paclitaxel improved the tissue microenvironment in which cancer cells grew, which changed include an increase in inflammatory monocytes and a decrease in cytotoxicity (121, 128, 129). Importantly, these changes in both primary and metastatic lung were dependent on *ATF3* stress-inducing genes in non-cancer host cells (127).

After that, exosomes produced by paclitaxel treated cancer cells could promote the release of exosomes from cancer cells, change the lung microenvironment and promote lung metastasis of breast cancer (130). Paclitaxel not only promoted the production of more exosomes in cancer cells, but also promoted the entry of annexin A6 (ANXA6) protein, a non-glycosylated polypeptide chain into exosomes by increasing calcium levels in cancer cells, which contributed to paclitaxel resistance in breast cancer (131). After ANXA6-carrying exosomes were transported to the lungs through blood circulation, they promoted the expression of chemokines in lung tissues and recruited mononuclear macrophages, which were the “agent provocateurs” that helped lung metastasis of breast cancer (132). Macrophages had been shown to play important roles in cancer metastasis. Macrophages were generally categorized into either of two functionally contrasting subtypes, namely classical activated M1 macrophages and alternatively activated M2 macrophages (133, 134). M2 macrophages promoted the occurrence and metastasis of tumor cells, inhibited T cell-mediated anti-tumor immune response, promoted tumor angiogenesis, and leaded to tumor progression (135). Both M1 and M2 macrophages had high degree of plasticity and thus could be converted into each other upon tumor microenvironment changes or therapeutic interventions (133).

In order to verify the effect of ANXA6, the researchers knocked out the ANXA6 gene in breast cancer cells and found that exosomes secreted by paclitaxel-induced cancer cells did not carry ANXA6 and no longer had the function of promoting lung metastasis of tumor (130). Blocking the metastasis promoting function of pulmonary mononuclear macrophages could also prevent paclitaxel-induced lung metastasis of breast cancer (130). In addition to paclitaxel, adriamycin also promoted the entry of ANXA6 into exosomes and facilitated lung metastasis of breast cancer (130). Paclitaxel and adriamycin could be said to be two completely different chemotherapy drugs, both of which promoted tumor metastasis through exosome and ANXA6, perhaps this effect of chemotherapy in promoting metastasis was widespread.

## Conclusions and Perspectives

The vast majority of cancer deaths were caused by metastasis (136). Current research suggested that tumor metastasis depended not only on the ability of tumor cells to invade themselves, but also on the formation of a metastatic niche. The construction of the metastatic microenvironment determined whether the tumor cells invading the circulatory system could attach, survived, proliferated and eventually formed metastatic cancer at the distant site of metastasis. Before metastasis of tumor cells, functional domestication of cells and matrix components in target organs of metastasis could be performed to form a microenvironment conducive to tumor cell colonization (137).

With the continuous development of the research on the metastatic microenvironment, results of more and more research on the metastatic lung microenvironment of breast cancer had been reported. Currently, there were in-depth studies on the neutrophils-dependent inflammatory microenvironment in lungs, and the inflammation induced oxidative stress response and promoted angiogenesis, which contributed to the metastatic growth of tumors (138). In addition, in the initial stage of inflammation, the activation of inflammatory factors also triggered vascular dilation, which increased the permeability of the vascular wall, and tumor cells exuded from the blood vessels and colonized as well as grew in the metastatic organs (66, 139). In addition, many cells or soluble factors in lungs were affected by the signals released by breast cancer cells, and then modified the lung microenvironment to make it suitable for the growth of breast cancer. Or cytokines in lungs microenvironment attracted chemotaxis, migration, and colonization of breast cancer cells, thereby promoting lung metastasis of breast cancer.

Although some progress had been made in the study of metastatic lung microenvironment of breast cancer, many mechanisms remained unclear due to the complexity and variability of metastatic microenvironment and high heterogeneity. Many questions needed to be further explored and solved: were these metastatic microenvironments induced by breast cancer or existed before the remodeling of the pulmonary microenvironment? How to optimize *in vitro* and *in vivo* experimental model to more accurately simulate the human lung microenvironment? In such a variable lung microenvironment, neutrophils, which were the most deeply studied, not only promoted tumor metastasis by inducing inflammatory lung microenvironment, but also stored nutrients for newly metastasis to lung tumor cells in advance to provide energy for their colonization and growth in lung. In addition, neutrophils could be directly measured in peripheral blood. Could corresponding kits be developed to detect markers of peripheral blood neutrophils, which were used as a monitoring indicator for the prognosis of breast cancer with lung metastasis? In sum, the research results of pulmonary microenvironment would be another major breakthrough in the treatment of lung metastasis of breast cancer and provided a new perspective for the suppression of lung metastasis of breast cancer.

The lung was a common site for metastasis of malignant tumors such as primary bladder cancer, breast cancer, colon cancer, kidney cancer, melanoma, ovarian cancer, pancreatic cancer, osteosarcoma, rectal cancer, gastric cancer, thyroid cancer and endometrial cancer (140). These findings above-mentioned encouraged the design of pre-clinical and clinical studies to examine the benefits of targeting the formation of pulmonary microenvironment in lung metastasis from bladder cancer, colon cancer, kidney cancer and so on, which might be effective in blocking metastatic relapse.

## Author Contributions

All authors listed have made a substantial, direct, and intellectual contribution to the work and approved it for publication.

## Funding

This study was supported by the grants from the Dongguan Social Science and Technology Development Project (201950715025192), the grants from the National Natural Science Foundation of China (81802918), the China Postdoctoral Science Foundation Grant (2019M660206), the Science and Technology Project of Guangdong Province (2019A1515011565, 2018A030310007), and the Science and Technology Project of Jiangmen (2020030103140008978, 2019030102430012905).

## Conflict of Interest

The authors declare that the research was conducted in the absence of any commercial or financial relationships that could be construed as a potential conflict of interest.

## Publisher’s Note

All claims expressed in this article are solely those of the authors and do not necessarily represent those of their affiliated organizations, or those of the publisher, the editors and the reviewers. Any product that may be evaluated in this article, or claim that may be made by its manufacturer, is not guaranteed or endorsed by the publisher.

## Abbreviation

LPS, lipopolysaccharide; LCN2, lipocalin 2; NADPH, nicotinamide adenine dinucleotide phosphate; NE, neutrophil elastase; MPO, myeloperoxidase; ALP, alkaline phosphatase; NETs, neutrophil extracellular traps; NF-κB, nuclear factor kappa B; CCL3, chemokine (C-C motif) ligand 3; ROS: reactive oxygen species; Tsp-1, thrombospondin-1; ALOX5, arachidonic acid 5-lipooxygenase; CXCL8, chemokine (C-X-C motif) ligand 8; DKK1, Dickkopf-1; PCP, planar cell polarity; CaMKII, calmodulin-dependent protein kinase II; TGF-β, transforming growth factor-β; PR3, proteinase 3; MAPK, mitogen-activated protein kinase; TLR3, Toll-like receptor 3; ER, estrogen receptor; ANGPTL4, angiopoietin-like protein 4; AT1, alveolar type 1; mCherry, monomeric Cherry red fluorescent protein; Foxp3, forkhead box protein 3; PHD, prolyl-hydroxylase; DAMPs, damp-associated molecular patterns; NLRP3, NOD-like receptor protein 3; MDSCs, marrow derived suppressor cells; CAIX, Carbonic anhydrase IX; MMTV, mammary tumor virus; TIE2, TEK tyrosine kinase; MENA, mammalian-enabled; ANXA6, annexin A6.
